# Single-center thorough evaluation and targeted treatment of globozoospermic men

**DOI:** 10.1007/s10815-021-02191-4

**Published:** 2021-04-20

**Authors:** Stephanie Cheung, Alessandra Parrella, Danielle Tavares, Derek Keating, Philip Xie, Zev Rosenwaks, Gianpiero D. Palermo

**Affiliations:** The Ronald O. Perelman and Claudia Cohen Center for Reproductive Medicine, Weill Cornell Medicine, 1305 York Avenue, Y720, New York, NY 10021 USA

**Keywords:** Globozoospermia, ICSI, Assisted oocyte activation, Phospholipase C zeta, Gene mutations, RNA expression, Proteomics

## Abstract

**Purpose:**

To characterize, by specific biomarkers and nucleic acid sequencing, the structural and genomic sperm characteristics of partial (PG) and complete globozoospermic (CG) men in order to identify the best reproductive treatment.

**Methods:**

We assessed spermatozoa from 14 consenting men ultrastructurally, as well as for histone content, sperm chromatin integrity, and sperm aneuploidy. Additional genomic, transcriptomic, and proteomic evaluations were carried out to further characterize the CG cohort. The presence of oocyte-activating sperm cytosolic factor (OASCF) was measured by a phospholipase C zeta (PLCζ) immunofluorescence assay. Couples were treated in subsequent cycles either by conventional ICSI or by ICSI with assisted gamete treatment (AGT) using calcium ionophore (Ionomycin, 19657, Sigma-Aldrich, Saint Louis, MO, USA).

**Results:**

Ultrastructural assessment confirmed complete acrosome deficiency in all spermatozoa from CG men. Histone content, sperm chromatin integrity, and sperm aneuploidy did not differ significantly between the PG (*n* = 4) and CG (*n* = 10) cohorts. PLCζ assessment indicated a positive presence of OASCF in 4 PG couples, who underwent subsequent ICSI cycles that yielded a 36.1% (43/119) fertilization with a 50% (2/4) clinical pregnancy and delivery rate. PLCζ assessment failed to detect OASCF for 8 CG patients who underwent 9 subsequent ICSI cycles with AGT, yielding a remarkable improvement of fertilization (39/97; 40.2%) (*P* = 0.00001). Embryo implantation (6/21; 28.6%) and clinical pregnancies (5/7; 71.4%) were also enhanced, resulting in 4 deliveries. Gene mutations (DPY19L2, SPATA16, PICK1) were identified in spermatozoa from CG patients. Additionally, CG patients unable to sustain a term pregnancy had gene mutations involved in zygote development (NLRP5) and postnatal development (BSX). CG patients who successfully sustained a pregnancy had a mutation (PIWIL1) related to sperm phenotype. PLCZ1 was both mutated and underexpressed in these CG patients, regardless of reproductive outcome.

**Conclusions:**

Sperm bioassays and genomic studies can be used to characterize this gamete’s capacity to support embryonic development and to tailor treatments maximizing reproductive outcome.

**Supplementary Information:**

The online version contains supplementary material available at 10.1007/s10815-021-02191-4.

## Introduction

Infertility is known to affect 15% of couples of reproductive age. Of these, 35% of cases are related to the female partner and 55% to the male partner, while the remaining 10% are of unknown origin [1]. Male infertility can be attributed to varicocele (26.4%), obstruction (15.1%), testicular failure (14.5%), cryptorchidism (14.3%), idiopathic (12.1%), or genetic factors (7.9%). Other minor contributors include infections (3%), hormones (2.3%), immunology (2.3%), ejaculatory dysfunction (1.2%), cancer (0.5%), and, as more recently recognized, systemic diseases (0.4%) [2]. All of these etiologies affect spermatozoa production and therefore compromise the typical semen parameters. Although concentration and kinetic characteristics of the male gamete are widely understood to be associated with its ability to fertilize an oocyte, sperm phenotype as evaluated by sperm morphology—particularly in relation to the sperm head—also indicates spermatozoa competence.

Among the phenotypes, there is a rare and severe type of teratozoospermia known as globozoospermia. This condition, first described in 1976 [3, 4], is prevalent in 0.1% of infertile men [5] and is characterized by round-headed spermatozoa with acrosomal hypoplasia/absence and cytoskeleton defects surrounding the nucleus [3, 6]. Two forms of globozoospermia have been described [7]. The most severe, defined as complete globozoospermia, or type I, entails the entirety of the spermatozoa accompanied by acrosomal absence [7]. The other form, known as partial globozoospermia, or type II, presents with a varied proportion of the spermatozoa bearing the round-headed hallmark and acrosomal cap dysmorphism [8]. Globozoospermic (GZ) men suffer from compromised spermiogenesis, mostly limited to the remodeling mechanism responsible for the absence of acrosome and cytoskeleton defects yielding the round nucleus. However, anomalies fluctuate and may include deficiency of the post-acrosomal sheath, detachment of the nuclear membrane, coiled tails, and cytoplasmic remnants at the midpiece [3, 8, 9].

The physiopathology of globozoospermia has been linked to multifactorial origins, with its basis stemming from inherited genetic traits [10] that tend to recur among male siblings [11]. The genetic constituent of this disorder results from recessive deletions and point mutations involving two main genes, *DPY19L2* [12] and *SPATA16* [13]. *DPY19L2*, expressed in the testis, is responsible for sperm head elongation and development of the acrosome during spermiogenesis, entailing a 200-Kb homozygous deletion encoded at the *DPY19L2* locus [14]. A *SPATA16* homozygous mutation (c.848G → A), also expressed in the testis, is responsible for the production of spermatozoa with the typical round head and absent acrosome. This is due to interference with the splicing site of exon 4, which codes for a tetratricopeptide repeat (TPR) domain [15].

Despite having normal parameters, spermatozoa of GZ men have peculiar head anomalies that render them incapable of interacting with and dispersing cumulus cells surrounding the oocyte. These spermatozoa are unable to bind to the ZP2 and ZP3 glycoproteins on the zona pellucida [16] and therefore cannot reach the perivitelline space. The only insemination method capable of overcoming this defect is intracytoplasmic sperm injection (ICSI), which bypasses the zona pellucida and oolemma altogether. Although ICSI has been proven to provide fertilization in patients with most forms of severe male factor infertility, its use still yields inconsistent results when treating globozoospermia [17]. This can be attributed to the complete lack of a putative oocyte-activating sperm cytosolic factor (OASCF), known as phospholipase C zeta (PLCζ), necessary to activate intracellular calcium (Ca^2+^) oscillations triggering oocyte activation [18, 19]. Indeed, GZ spermatozoa not only suffer from acrosomal absence, but they also have concurrent impaired development of the perinuclear theca where the sperm cytosolic factors reside. Therefore, these gametes require the exposure of the injected oocyte to a chemical activating agent such as calcium ionophore to artificially induce the calcium cascade. The use of oocyte activation with ICSI has been shown to achieve fertilization and successful pregnancies, even in patients with the most severe cases of globozoospermia [5]. However, its application has recently been shown to be necessary only in cases with a confirmed sperm-related, oocyte-activating deficiency [20].

Although there is significant literature on globozoospermia, few studies have compared the two forms to determine the best method of treatment for each. For instance, an assessment of 12 GZ men identified 3 underexpressed genes when compared to a fertile cohort. And although a higher fertilization and clinical pregnancy rate following ICSI with oocyte activation was observed, the study cohort was limited to complete GZ men; patients with the partial GZ phenotype were not included [21]. A separate multicenter study of 34 patients further confirmed the effectiveness of ICSI with oocyte activation in GZ patients according to their DPY19L2 mutation status [22]. No other sperm function assays were performed to compare partial (PG) and complete globozoospermic (CG) groups. However, the genetic assessment failed to include additional genes associated with globozoospermia, such as SPATA16 and PICK1 [23, 24]. The impact of mutations on gene expression was also not addressed. Although gene mutations may be present, the transcriptome may not necessarily be impaired. Furthermore, different steps of the gene’s expression and protein activity may be altered depending on the type and location of the mutation [25].

In the current study, we aim to characterize, by specific biomarkers and nucleic acid sequencing, the structural and genomic sperm characteristics of PG and CG men in order to identify the best reproductive treatment. This assessment represents the largest comparison of PG and CG men in a single assisted reproductive technology (ART) center. Specimens from these individuals were screened by multiple gamete functional assays to measure the presence of OASCF and ultrastructural features. Genomic assessments of histone content, chromatin integrity, and aneuploidy were also performed. According to test results, couples were categorized by the degree of globozoospermia and treated according to OASCF content. Only couples with absent OASCF were treated by ICSI with assisted gamete treatment (AGT), where both the spermatozoa and oocytes were exposed to calcium ionophore [20]. Clinical outcomes were compared between the two globozoospermia forms and their respective treatment protocols. In addition, transcriptomic analyses were carried out to identify genes involved in the peculiar spermiogenic process of these GZ men.

## Materials and methods

### Inclusion criteria and study design

Over the course of 8 years, a total of 14 couples (maternal age, 33.7±3 years; paternal age, 36.1±3 years) were enrolled in this study. Patients were referred to our center after presenting with morphologically round-headed spermatozoa and a history of poor or absent fertilization with ART elsewhere.

Following standard semen analysis [26], spermatozoa from each patient were assessed ultrastructurally, as well as for histone content, sperm chromatin integrity, and aneuploidy. Patients were then allocated to two groups based on the proportion of round-headed spermatozoa. Those who exhibited 100% of the round-headed morphology were diagnosed as CG, while those with a large fraction of spermatozoa exhibiting round-headed morphology were considered PG. Additional genetic, transcriptomic, and proteomic analyses were performed on spermatozoa from consenting men (*n* = 3), in comparison to a fertile donor control (*n* = 3), to further characterize the CG type.

**Fig. 1:**
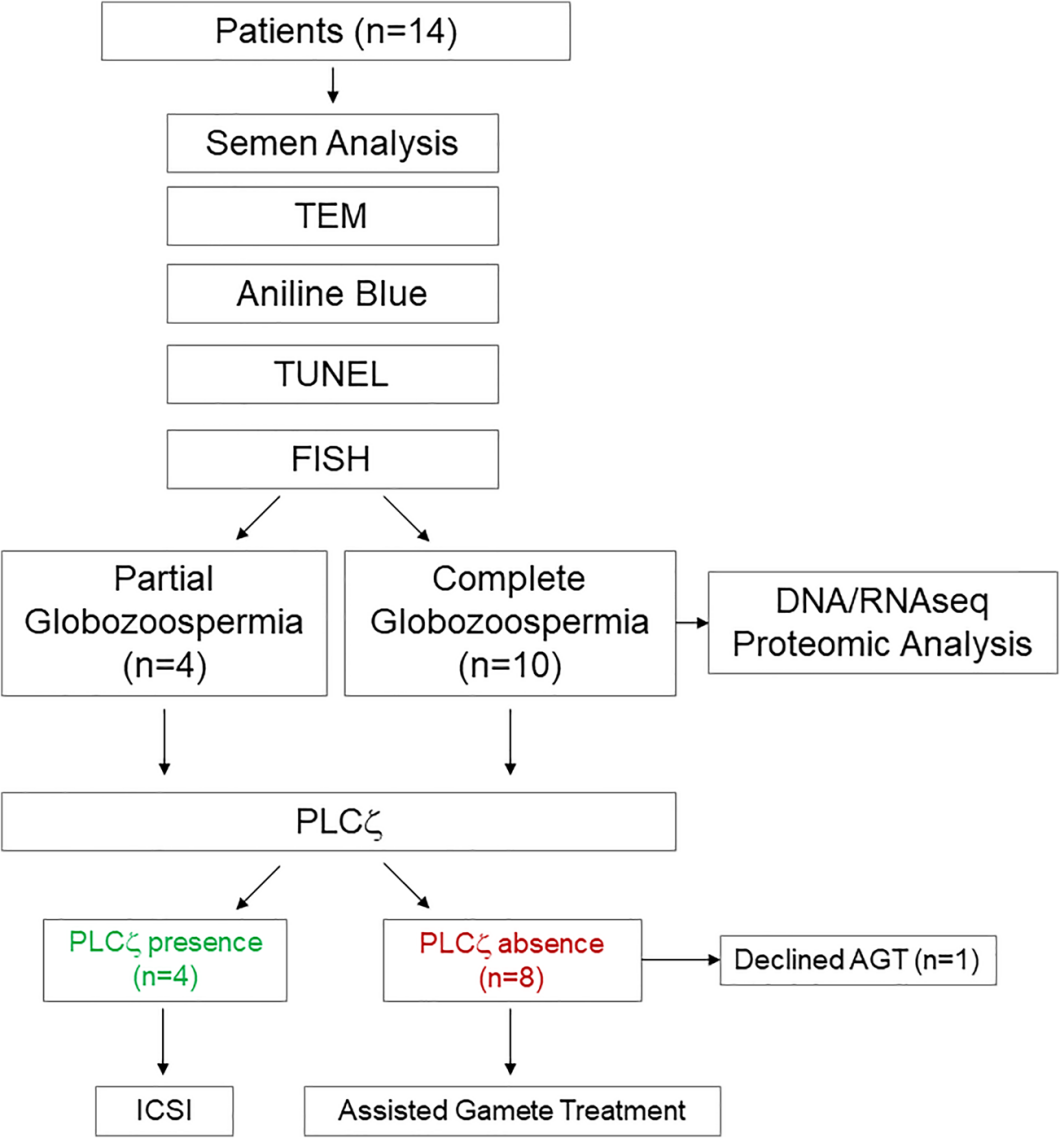
A total of 14 couples were enrolled in this study. Following standard semen analysis, spermatozoa from each patient were assessed ultrastructurally, as well as for histone content, sperm chromatin integrity, and aneuploidy. Patients were then allocated to two groups based on the proportion of round-headed spermatozoa. Those who exhibited 100% of the round-headed morphology were diagnosed as complete globozoospermic (CG), while those with a large fraction of spermatozoa exhibiting round-headed morphology were considered partial globozoospermic (PG). Additional genetics and transcriptomic analyses were performed on the spermatozoa from consenting men (*n* = 3) to further characterize the CG type. Of the 14 couples, 2 eventually elected to be treated elsewhere or not pursue infertility treatment. We then screened patients for OASCF by PLCζ assessment. Couples with a normal OASCF who were continuing at our center (*n* = 4) were treated with conventional ICSI, while consenting couples with an OASCF deficiency (*n* = 7) were treated by ICSI with AGT

Although 2 couples eventually elected not to pursue infertility treatment, we screened all patients for OASCF by PLCζ assessment. Couples with a normal OASCF who were continuing treatment at our center (*n* = 4) underwent conventional ICSI, while consenting couples with an OASCF deficiency (*n* = 7) were treated by ICSI with AGT in their subsequent cycles (Fig. 1) [20].

This study, performed at a single academic center, was approved by the Institutional Review Board of New York Presbyterian Hospital-Weill Cornell Medicine (IRB 0712009553 and 1006011085), and all participants were appropriately counseled before providing consent. We do not have any potential conflict of interest to disclose.

### Spermatozoa collection and preparation

Fresh ejaculates were collected by masturbation following 2–5 days of abstinence. Semen was allowed to liquefy at 37 °C for at least 15 min. Initial analysis of sperm concentration and motility was conducted on 5 μl of specimen using a Makler® sperm-counting chamber (Sefi Medical Instruments, Ltd., Haifa, Israel). Sperm morphology was assessed on Testsimplets® pre-stained slides (Waldeck GbmH, Münster, Germany) at 1000× magnification. Aliquots of the specimen were then used for the additional sperm bioassays.

### Ultrastructural sperm assessment

Transmission electron microscopy (TEM) was performed on at least 200 spermatozoa to assess organelles including acrosomes, nuclei, centrioles, and the microtubular arrangement in flagella [5]. Post-centrifugation cell pellets were resuspended uniformly in cold fixative containing 2.5% glutaraldehyde, 4% paraformaldehyde, and 0.02% picric acid in 0.1 M sodium cacodylate buffer. After minimal 1-h fixation at 4 °C, cell pellets were collected and washed thrice in buffer and treated by secondary fixative solution containing 1% osmium tetroxide and 1.5% potassium ferricyanide [27]. Fixed samples were then exposed to uranyl acetate for *en bloc* staining and dehydrated sequentially in increasing concentrations of ethanol. Dehydrated specimens were infiltrated and embedded in Spurr’s resin, and then sliced by ultramicrotome to 100-nm sections. These sections were then viewed by JEOL-1400 electron microscope (JEOL USA, Inc., Peabody, MA, USA) at 300,000× magnification, where a sperm ultrastructure tomography was generated from the reflection of electrons by a 120-kV electron beam transmitted through the section. Slides were observed to assess chromatin integrity as well as acrosomal and perinuclear theca structural and centrosomal integrity.

### Histone content assay

Histone content assay using aniline blue staining was carried out to assess the nuclear maturity status of the spermatozoa. Slides were prepared by smearing raw specimen, air-drying, and fixing in 4% paraformaldehyde solution for 10 min, and then staining for 5 min in 5% aqueous aniline blue solution (pH 3.5) [28, 29]. Spermatozoa were analyzed under bright-field illumination at 1000× magnification. Those with immature nuclear chromatin, characterized by high content of histone proteins, stained dark blue. Those with mature chromatin, rich in protamines, did not uptake the stain. A minimum of 200 spermatozoa were assessed per patient, with a normal threshold of ≤20% stained spermatozoa [30].

### Sperm chromatin fragmentation assay

The integrity of sperm chromatin fragmentation (SCF) was measured by terminal deoxynucleotidyl transferase–mediated deoxyuridine triphosphate-fluorescein nick-end labeling (TUNEL) assay. The protocol used for this test has been previously described [17]. In brief, after liquefaction of raw sample at 37 °C for 15 min, approximately 5 μl of specimen was smeared on a slide and left to dry overnight. The slides were fixed with 4% paraformaldehyde for 1 h and, after washing thrice with PBS, left to dry overnight. Slides were then permeabilized in a solution containing 0.1% Triton X-100 and 0.1% sodium citrate in PBS and washed with PBS. The reagents from a commercially available kit (in situ cell death detection kit; Roche Diagnostics, Rotkreuz, Switzerland) were applied to the slides, which were incubated in a humidified chamber at 37 °C for 1 h. To visualize the sperm nuclei under a fluorescent microscope, DAPI Antifade (Millipore, Temecula, CA, USA) was added to the slides. At least 500 spermatozoa per patient were counted, with a normal threshold of ≤15% chromatin fragmentation [31].

### Sperm aneuploidy assessment

Ejaculated specimens were prepared for FISH aneuploidy assessment as previously described [32]. Five microliters of semen samples was smeared on glass slides and air-dried overnight. Slides were fixed in Carnoy’s fixative (3:1 methanol:acetic acid) at room temperature and subsequently placed in a 37 °C slide moat overnight. Sperm nuclei decondensation was carried out by incubating slides in 5 mmol/L dithiothreitol (DTT; Sigma Chemical Co., St. Louis, MO) in 0.1 M tris(hydroxymethyl)aminomethane (Trizma HCl; Sigma Chemical Co.), and then by 3 M sodium chloride and 300 mM tri-sodium citrate dehydrate (2× standard saline citrate; Vysis, Downers Grove, IL), pH 7.0. The decondensed slides were hybridized with fluorescent probes specific for chromosomes X, Y, 13, 15, 16, 17, 18, 21, and 22. Sperm nuclei were then counterstained with 4′,6-diamino-2-phenylindole (DAPI; Millipore, Temecula, CA, USA). A minimum of 1000 spermatozoa per specimen were analyzed under an Olympus BX61 fluorescent microscope at 1000×. Incidences of disomy, nullisomy, and diploidy were recorded using a computerized system (Applied Imaging, CytoVysion v3.93.2), with a <1.6% normal threshold [32].

### OASCF assessment

The presence of OASCF was assessed by detection of PLCζ. Sperm specimens were smeared on glass slides, fixed with recently prepared 4% paraformaldehyde solution/PBS for 20 min, and washed with 0.1% Tween 20/PBS for 5 min. Slides were permeabilized with 1% Triton X-100/PBS for 1 h at room temperature. To block unspecific binding sites, 5% of normal goat serum/PBS was added on the specimen for 1 h at 37 °C. The specimens were then incubated overnight with anti-rabbit PLCζ antibody (pab0367, Covalab, Bron, France) in 5% normal goat serum/PBS at 4 °C. After rinsing in PBS, slides with specimens were incubated with FITC-coated goat anti-rabbit IgG (ab150077, Abcam, Cambridge, UK), which cross-reacts with human PLCζ. Lastly, spermatozoa were counterstained with 10 μg/ml 4′,6-diamino-2-phenylindole (DAPI/Antifade Solution, Millipore, Temecula, CA, USA) and visualized under a fluorescent microscope to assess the percentage of spermatozoa exhibiting PLCζ fluorescence in the acrosomal, equatorial, and post-acrosomal regions of the sperm head. At least 200 spermatozoa were counted for each specimen. By assessing a large number of patients for PLCζ, we found that those with less than 30% PLCζ yielded consistently low or unobtainable fertilization. Therefore, the threshold of 30% was adopted [20].

### Stimulation protocols and oocyte retrieval

Superovulation protocols were determined based on patient age, weight, antral follicular count, serum anti-Müllerian hormone (AMH) level, and historical treatment. Gonadotropins (Menopur, Ferring Pharmaceuticals Inc., Parsippany, NJ, USA; Gonal F, EMD Serono, Geneva, Switzerland; and/or Follistim, Merck, Kenilworth NJ, USA) were administered daily, while pituitary gland functions were suppressed by GnRH antagonist (Cetrotide, EMD Serono Inc., Rockland, MA, USA; or Ganirelix acetate, Merck, Kenilworth, NJ, USA) or GnRH agonist (leuprolide acetate, Abbott Laboratories, Chicago, IL, USA). When the 2 leading follicles reached a diameter of 17 mm, human chorionic gonadotropin (hCG, Ovidrel, EMD Serono) was administered for triggering. Transvaginal oocyte retrieval was performed 35–37 h after hCG trigger.

Retrieved cumulus–oocyte complexes (COCs) were incubated for 3–4 h, to allow for ooplasmic maturation [33, 34]. Before micromanipulation, denudation of COCs was carried out by exposure to 40 IU/mL hyaluronidase (Cumulase, Halozyme Therapeutics, Inc. San Diego, CA). To facilitate denudation and prevent overexposure to hyaluronidase, COCs were aspirated repetitively in a stripping pipette with an inner diameter of 200 μm (Origio, Målov, Denmark). Denuded oocytes were washed twice in early embryo culture medium (modified medium based on G1 and G2 components; Vitrolife, Göteborg, Sweden) [35, 36]. Resulting oocytes were then examined under an inverted microscope (TE2000U, Nikon USA, Melville, New York, USA) to assess oocyte maturational stage [33]. Only oocytes displaying the first polar body (PB) were considered metaphase II and used for ICSI.

### Assisted gamete treatment

AGT was carried out by exposing spermatozoa and post-ICSI oocytes to calcium ionophore (19657, Sigma-Aldrich, Saint Louis, MO, USA) as previously described [20]. Before ICSI injection, ejaculated spermatozoa processed by density gradient or centrifugation were briefly exposed to 50 μM calcium ionophore in G-MOPS™ media (Vitrolife, Göteborg, Sweden) enriched with human serum albumin (G-MM™, Vitrolife, Göteborg, Sweden). During the ICSI procedure, spermatozoa were aspirated individually from the media drop containing calcium ionophore and immobilized in a separate polyvinylpyrrolidone (PVP) drop. Then, a small portion of calcium ionophore was aspirated into the micropipette and injected into the oocyte with the spermatozoa. Next, post-ICSI oocytes were exposed to 50 μM calcium ionophore in G-1 medium (Vitrolife, Göteborg, Sweden) for 10 min at 37 °C, and then rinsed and placed in fresh G-1 medium [20].

### Preimplantation development, embryo transfer, and pregnancy outcomes

Post-ICSI oocytes were loaded into EmbryoScope time-lapse incubators (Vitrolife, Göteborg, Sweden). Fertilization assessment was carried out 16–18 h after ICSI by evaluating zygotes for the presence of two distinct pronuclei and extrusion of the second PB [37]. Embryo morphokinetic parameters were recorded and evaluated.

Embryo transfer was performed on day 3 or 5 at the cleavage or blastocyst stage, respectively. Patients who elected to have fresh embryo transfer received 50 mg progesterone daily through intramuscular injection 24-hpost-retrieval. βhCG levels were measured 14 days after retrieval. Clinical pregnancy was defined by the presence of a fetal heartbeat detected through ultrasound at 7-week gestation. Delivery method and newborn health were obtained from obstetrical and pediatric records.

### Genomic analysis by NGS

Sperm specimens were processed by centrifugation at 600 *g* for 10 min in human tubal fluid medium (HTF; Irvine Scientific, Santa Ana, CA). After adjusting the concentration to 500 sperm cells/mL for each sample, DNA extraction and amplification were carried out as previously described, with the use of a commercial kit (Repli-G Single Cell; Qiagen, Hilden, Germany) [32]. Amplified DNA specimens were submitted for quality control testing, where a DNA concentration of 417.7 ± 283 ng/μl and quality of 1.7 ± 0.1 was confirmed. Specimens were then sent to an external facility (Genewiz, Inc.; South Plainfield, NJ) where they were processed by 150-bp paired-end sequencing on an Illumina HiSeq 2500. Reads were trimmed to remove nucleotides with poor quality (error rate <0.01) and aligned to the hg20 reference genome using CLC Genomics Server 9.0. After quality assessments, QPCR was carried out to obtain high quality exome coverage of at least 90% (Agilent SureSelect Human All Exon V6). Detected copy number variants were further annotated for gene mutation analysis in comparison with a fertile sperm donor control. Genes were considered duplicated or deleted when the read depth was greater than or less than 1.5 times the median depth of the control for ≥70% exons in the gene [32].

### Transcriptome analysis

A total of 4 to 5 × 10^6^/ml sperm cells were prepared for RNA sequencing as previously described [32]. In brief, total RNA was isolated and purified using an RNeasy Mini Kit spin column (RNeasy; Qiagen, Hilden, Germany). Nucleic acid quantification was performed by an Agilent 2100 bioanalyzer to determine the RNA integrity number (RIN), while spermatozoal RNA concentration was determined by a NanoDrop spectrophotometer and confirmed by Quibit RNA assay. After library prep (NEBNext Ultra RNA Library Prep kit, New England BioLabs Inc., Ipswich, MA), ribosomal RNAs were isolated from total RNA by rRNA depletion (Ribo-Zero Gold rRNA Removal kit, Illumina, San Diego, CA). Sequencing was performed (NextSeq500; Illumina, San Diego, CA) at a pilot paired-end 36 bp before being expanded to 50–60-M reads at 2 × 75 bp. Sequenced reads were trimmed to remove low-quality bases using Trimmomatic v.0.36, and then mapped to the hg20 reference genome by CLC Genomics Server 9.0. For differential expression analysis, raw read counts were uploaded according to the DESeq2 v1.23.1 (LGPL, Bioconductor) pipeline. After data normalization, gene expression comparison was performed. To avoid over- or under-representing fragments per kilobase of transcript per million mapped reads (FPKM), an algorithm by edgeR (LGPL; Bioconductor) and CONTRA was implemented following the DESeq2 expression analysis to overcome experimental conditions such as fragmentation [32].

### Proteomic analysis

Sperm specimens were centrifuged after 1:1 dilution in PBS at 600 g for 10 min to remove the seminal fluid. The pellets were digested in a trypsin solution overnight at 37 °C following reduction with 5 mM DTT and 14 mM alkylation with iodoacetamide. The digested samples were vacuum centrifuged to near dryness and desalted with a Sep-Pak column.

A Thermo Fisher Scientific EASY-nLC 1000, coupled with a Fusion Lumos mass spectrometer (Thermo Fisher Scientific), was used. Gradient separation was conducted using 0.1% formic acid in water (buffer A) and 0.1% formic acid in acetonitrile (buffer B) as mobile phases (Cox and Mann, 2008). Peptide separation was performed using a 75 μm × 15 cm chromatography column (ReproSil-Pur C18-AQ, 3 μm, Dr. Maisch GmbH, Germany) with gradients of 3–28% and 28–80% buffer B over 50 and 10 min, respectively, at a flow rate of 300 nL/min. The Fusion Lumos mass spectrometer was operated in data-dependent mode. Full mass spectrometry (MS) scans were acquired on the Orbitrap mass analyzer over a range of 300–1500 m/z with 120,000 resolution at 200 m/z. The 20 most abundant precursors with charge states between 2 and 5 were selected with an isolation window of 1.4 Thomsons by the quadrupole and fragmented by collision induced dissociation with normalized collision energy of 35 in the ion trap. Tandem mass spectrometry (MS/MS) scans were acquired in the ion trap. The automatic gain control target value was 4e5 for full scans and 20,000 for MS/MS scans respectively, and the maximum ion injection time was 50 ms for both.

Samples were processed and analyzed individually in comparison to a fertile control. Protein identification was conducted using the MaxQuant (Cox and Mann, 2008) computational proteomics platform version 1.5.5.1 (Max Planck Institute, Munich, Germany). The UniProt human protein database was searched using the fragmentation spectra. To query the database, oxidation of methionine and protein N-terminal acetylation were used as variable modifications. Precursor mass tolerance was fixed at 7 ppm while the fragment mass tolerance was set at 20 ppm. Both peptide and protein identifications were filtered at 1% false discovery rate based on decoy search using a database with the protein sequences reversed. Fold change values were obtained by obtaining the difference between the intensity values of the experimental and control groups, which were then log2 transformed and normalized.

### Data analysis

Student’s *t*-test was used to analyze continuous variables that were reported as mean ± standard deviation. Fisher’s exact test (Jandel Scientific, San Rafael, CA) and Friedman’s chi-square test were performed to test the relationship between fertilization, clinical pregnancy rate, and pregnancy loss. A *P* value of <0.05 was considered statistically significant.

Copy number variant (CNV) assessment was carried out using CLC Genomics Server 9.0 modules including the NGS Core Tools/Mapping and Re-sequence analysis. RNAseq data analysis for differential gene expression was performed using DESeq2 v1.23.1 (LGPL, Bioconductor) and calculated in FPKM. Statistical thresholds of *P* < 0.0005 for significance and *Q* < 0.05 threshold for false positive discovery were used.

## Results

### Sperm function, ultrastructure, and genomic characterization of globozoospermia

This study, which spans 8 years, describes 14 men (25–41 years old) with globozoospermia. The patients’ semen parameters are presented in Table 1. According to their morphological assessments, 4 patients were considered PG while 10 were categorized as CG.

**Table 1:** Couples’ demographics, gamete characteristics, and outcomes for previous ICSI cycles

Number of (%)	Total
Couples	14
Female age	33.7 ± 3
Male age	36.1 ± 3
Semen parameters	
Concentration (×10^6^/mL)	36.0 ± 44
Motility (%)	20.3 ± 20
Normal morphology (%)	0.2 ± 0.4
Cycles	21
Injected oocytes (M ± SD)	12.7 ± 9
Fertilization (%)	35/268 (13.1)
Cycles with embryo transfer*	11
Embryos transferred per cycle (M ± SD)	1.7 ± 1
Implantation (%)	1/19 (5.2)
Clinical pregnancy per cycle (%)	1/11 (9.0)
Clinical pregnancy per patient (%)	1/8 (12.5)
Delivered per patient (%)	0 (0)
Pregnancy loss per patient (%)	1 (100)

^*^Embryo transfer was performed on day 3 or 5 at the cleavage or blastocyst stage, respectively

A series of tests was carried out to evaluate the functional, structural, and genomic characteristics of the spermatozoa. Sperm function tests included assessing structural details by TEM, quantification of histone content, sperm DNA fragmentation, and sperm aneuploidy (Table 2).

**Table 2:** Sperm function, structural, and genomic characteristics

Parameters % (mean ± SD)	All (*n* = 14)	Partial (*n* = 4)	Complete (*n* = 10)	*P* value
OASCF	27.0 ± 22	34 ± 4	7.4 ± 4	0.001
Abnormal TEM	98.8 ± 1	98 ± 1	100 ± 0	0.01
Residual histones	46.9 ± 21	40 ± 19	53.8 ± 24	NS
DNA fragmentation	18.1 ± 8	16.3 ± 2	19.5 ± 12	NS
FISH aneuploidy	4.3 ± 5	0.9 ± 0	5.5 ± 5	NS

*P* < 0.05 was considered to be statistically significant

TEM revealed that most of the cells (98.0 ± 1%) observed from the PG group displayed a characteristic round-headed shape, often with concurrent absence of the acrosomal cap, dispersed chromatin content indicating a poor nuclear compaction, and a central nuclear inclusion. These abnormalities ranged from 97 to 100%. As expected, TEM confirmed the characteristic round-headed shape and acrosomal absence in 100% of the spermatozoa assessed from the CG group. Interestingly, centrosome and capitulum structures were intact (Supplementary Figure 1).

We attempted to characterize the type of globozoospermia of our study population by morphological and ultrastructural assessment. Semen parameters did not differ between the two forms of globozoospermia except, as expected, for the sperm morphology, which was more impaired in the CG cohort (*P* = 0.003). Similarly, the phenotype of the different sperm components indicated that head defects were the most prevalent and severe in the CG group, mainly due to the round-headed morphology with absent acrosomes (Supplementary Table 1). In addition, all spermatozoa of CG men had round-shaped heads with complete acrosome deficiency (*P* < 0.05).

Chromatin condensation abnormalities within the sperm nucleus appeared in both GZ types, fluctuating between 23 and 89% of the spermatozoa maintaining residual histones. The CG group had a higher frequency of spermatozoa with an increase in histone residues when compared to the PG group, albeit without significant difference. This observation also corroborated the poor compaction of chromatin seen with ultrastructural analysis.

The DNA fragmentation assay revealed only a mild increase in SCF in all GZ patients, ranging from 11.8 to 37.3%. A higher SCF was observed in the CG cohort, but without reaching statistical significance.

FISH assessment for the PG cohort indicated an overall sperm aneuploidy of 2.1 ± 2%. Targeted chromosome analysis revealed that total aneuploidy was mainly represented by autosomal disomy for chromosomes 18 and 22. FISH analysis showed a higher total aneuploidy at 5.5 ± 1% in the CG cohort as well, which was characterized by a high incidence of autosomal disomy for chromosomes 13, 15, 16, and 18. In addition, the occurrence of nullisomy was particularly high in this cohort (*P* < 0.05).

The presence of OASCF ranged from 2.5 to 55%. The CG cohort lacked the characteristic band on the sperm head in most of the cells and showed an extremely reduced level of PLCζ detectable in the post-acrosomal region of only 7.4% of the spermatozoa evaluated, remarkably lower than the PG group (*P* < 0.001) (Supplementary Figure 2, Table 1).

### ICSI outcomes

A total of 14 couples were included in our assessment (maternal age, 33.1 ± 3 years; paternal age, 35.7 ± 3 years). In 21 previous ICSI cycles, an average of 12.7 injected oocytes yielded a fertilization rate of 13.1% (35/268), an implantation rate of 5.2% (1/19), and a clinical pregnancy rate of 12.5% (1/8) that resulted in pregnancy loss (Table 1). Although 2 of these couples elected to be treated elsewhere or not to pursue infertility treatment, the remaining 12 opted to undergo subsequent ICSI cycles at our center. Couples were screened for the presence of OASCF by a PLCζ assay to determine whether AGT was necessary.

PLCζ assessment showed a positive presence of OASCF in 4 couples. These couples, who were also diagnosed with partial globozoospermia, underwent 4 previous cycles in which an average of 11.7 oocytes were injected, with a fertilization rate of 38.2% (18/47). Although all 4 couples underwent embryo replacement, none achieved a clinical pregnancy. These couples then underwent subsequent cycles at our center. An average of 14.8 oocytes were injected in 8 ICSI cycles, yielding a fertilization rate of 36.1% (43/119). This time, all 4 couples underwent embryo replacement that yielded clinical pregnancy and delivery rates of 50% (2/4) (Table 3).

**Table 3:** ICSI outcomes of couples with the presence of OASCF in comparison to their historical cycles

Number of (%)	Treatment		*P* value
	Previous cycles	Subsequent ICSI	
Couples	4		
Female age	32.2 ± 1	33.0 ± 1	NS
Male age	33.5 ± 2	34.0 ± 2	NS
Cycles	4	8	–
Injected oocytes (M ± SD)	11.7 ± 5	14.8 ± 4	NS
Fertilization (%)	18/47 (38.2)	43/119 (36.1)	NS
Cycles with embryo transfer	4	8	–
Embryos transferred per cycle (M ± SD)	2.7 ± 1	2.5 ± 1	NS
Implantation (%)	0/11 (0)	4/20 (20)	NS
Clinical pregnancy per patient (%)	0/4 (0)	2/4 (50)	NS
Deliveries (%)	–	2 (100)	NS

*NS*, nonsignificant

OASCF assessment failed to detect the presence of PLCζ in the spermatozoa from 8 CG patients. In their previous cycles, an average of 15.5 oocytes were injected, resulting in a fertilization rate of only 5.7% (8/140). Furthermore, none of the patients achieved implantation. Although one of the couples with confirmed absence of OASCF underwent subsequent ICSI cycles, they declined AGT. Thus, their resulting fertilization rate remained low, at 6.1% (3/49), and they did not achieve a pregnancy. The remaining 7 CG couples consented to AGT, and their subsequent ICSI cycles yielded a significantly higher fertilization rate of 40.2% (39/97, *P* < 0.0001). AGT also yielded more embryos transferred (*P =* 0.005) as well as higher implantation (28.6%, 6/21), clinical pregnancy (71.4%, 5/7), and delivery rates (80.0%, 4/5) (Table 4).

**Table 4:** ICSI outcomes of couples with the absence of OASCF in comparison to their historical cycles

Number of (%)	Treatment		*P* value
	Previous cycles	Subsequent ICSI + AGT	
Couples	7		
Female age	34.4 ± 8		
Male age	35.5 ± 7		
Cycles	9	9	–
Injected oocytes (M ± SD)	15.5 ± 14	10.7 ± 9	NS
Fertilization (%)	8/140 (5.7)	39/97 (40.2)	0.00001
Cycles with embryo transfer	4	9	–
Embryos transferred per cycle (M ± SD)	0.8 ± 1	2.3 ± 1	0.005
Implantation (%)	0/5 (0)	6/21 (28.6)	NS
Clinical pregnancy per patient (%)	0/7 (0)	5/7 (71.4)	NS
Deliveries (%)	0 (0)	4 (80)	NS
Pregnancy loss (%)	–	1 (20)	NS

*P *< 0.05 was considered to be statistically significant; *NS*, nonsignificant

### Gene profiling of men with complete globozoospermia

To confirm the presence of aneuploidy and identify germline mutations that would fail detection by somatic mutation analysis, we carried out whole-exome sequencing on the spermatozoa of 3 consenting men from the CG cohort. These men differed in their cycle outcomes: CG1 failed to achieve implantation, CG2 achieved implantation but had pregnancy loss at 8 weeks, and CG3 delivered healthy twins.

CNV analysis carried out on the gametes from these men yielded an overall aneuploidy rate of 8.2%. This is remarkably higher than when assessed only by 9-chromosome FISH (*P* < 0.05). Moreover, when we compared the gene mutations in these men to those of a fertile control, we identified single-nucleotide deletions in the DPY19L2, SPATA16, and PICK1 genes. These point mutations, highly represented in the GZ population, confirmed a complete absence of the subacrosomal perinuclear theca. In addition, we found that all three patients carried a single-nucleotide insertion on PIWIL1, associated with late spermiogenesis.

We then performed a gene mutation analysis for these patients according to their reproductive outcomes. Patient CG1 was found to have a mutation on NLRP5, a gene that plays an essential role in zygote progression beyond the first embryonic cell divisions. In patient CG2, who had a pregnancy loss at 8 weeks, exome sequencing revealed a heterozygous deletion on BSX, a gene related to postnatal development. Genetic assessment for patient CG3, who successfully delivered twins, identified a single-nucleotide insertion on PIWIL1, while genes related to embryogenesis were normal. PLCZ1 was found mutated in all 3 CG patients, regardless of their reproductive outcome.

Overall, identifying these relevant gene mutations confirmed that the phenotype and reproductive potential of the gametes was impaired. This underscored the importance of performing a precise genetic assessment to determine the best counselling and treatment option for GZ patients.

### Transcriptomic profiling of men with complete globozoospermia

To further delineate the epigenetic profile of these 3 CG individuals, RNA sequencing was carried out. Out of the 23,260 total genes assessed, 1866 significantly imbalanced genes were identified among the 3 patients (Supplementary Figure 3). From those, 111 were reproductive genes of which 34 were underexpressed. Most of the imbalanced genes (*n* = 1839) were found in men unable to sustain a pregnancy (CG1 and CG2), including 108 genes involved in reproductive processes. This transcriptomic analysis by RNAseq confirmed that in CG1, *DPY19L2*, *SPATA16*, and *PICK1* were all underexpressed, while in CG2, only *PICK1* was underexpressed. As for CG3, *DPY19L2* and *PICK1* were both significantly overexpressed, further confirming the malfunction of these two genes but apparently only limited to the gamete phenotype.

Looking more specifically at patient CG1, who failed to achieve implantation, 19 underexpressed genes were identified of which 2 were related to embryo development and implantation (*NEK2*, *STC2*) (Supplementary Table 2). Other relevant underexpressed genes include those that play a role in spermatogenesis (*KLHDC3*, *KLHL10*, *MORN2*, *PAPPA*, and *UBR2*), spermatozoa maturation (*ADAM21*, *CNBD2*, *SPEM1*, and *TXNRD3*), DNA condensation (*H1FNT*, *NEK2*, and *RNF8*), and fertilization capacity (*CALR3*, *HSPA1L*, and *IQCF1*), as well as an additional gene known to be commonly found in GZ patients (*MFSD14A*). Interestingly, all genes were located exclusively on the autosomes.

The analysis of patient CG2, who was able to achieve implantation but had pregnancy loss, identified 13 underexpressed genes. Three of these genes are involved in embryo development (*CBX2*, *RMI1*, and *TGFB3*). The remaining imbalanced genes play key roles in spermatogenesis (*CSF1*, *NAMPT*, *SEMG1*, *SEMG2*, and *SMCP*) and DNA-binding transcription factor activity (*FOXL2*, *GATA1*, and *LHX9*) (Supplementary Table 3). Interestingly, all these gene imbalances were located on autosomes with the exception of *GATA1*, which was on the X chromosome.

As for patient CG3, who was able to sustain a term pregnancy, only 2 underexpressed genes, *HAS2* (Chr 8) and *TGFB2* (Chr 19), both with generic functions involving cell adhesion, migration, and signaling receptor binding, were identified.

We also found common gene imbalances between CG1 and CG2, who both failed to sustain a term pregnancy (Supplementary Table 4). Out of the 14 genes identified, 8 were dedicated to generic functions. The remaining 6 genes, however, were involved in reproductive functions and were all overexpressed in both patients, except *NEK2* and *UBR2*, which were underexpressed only in CG1, the patient unable to achieve implantation. *PLCZ1*, however, was underexpressed in all 3 CG patients regardless of reproductive outcome. Not only did this confirm our DNAseq results, but it was corroborated by a preliminary proteomic analysis.

We were then interested in identifying gene imbalances common in all three CG men, and found only 1 gene, *LYL1*, that was underexpressed in CG1 and CG2 but overexpressed in CG3, which may be involved in placentation.

### Proteomic profiling of men with complete globozoospermia

We also carried out a preliminary proteomics analysis on these 3 CG men, to explore the protein expression patterns impacted by the gene mutations and imbalances (Supplementary Table 6). Overall, we identified 122 proteins that were upregulated, and 354 that were downregulated. This transcriptomics data was then compared to the genomic profiling of these men, distinguishing 6 genes that, in addition to mutations, appeared to be affected on an RNA and protein level. HIST1H1B (nucleosomal DNA binding), BAG5 (cell apoptosis), DPY19L2 and SPACA4 (acrosome development) proteins all exhibited a significant downregulation that was also reflected by the genes’ underexpression and deletions. In contrast, RCC1 and H1FNT, involved in chromosome condensation, chromatin binding, and nucleosomal DNA binding during spermatogenesis, showed a significant downregulation and underexpression at the protein and RNA level, respectively, despite the presence of single-nucleotide insertions.

## Discussion

Although globozoospermia is a relatively rare form of male infertility, its peculiar phenotype has severe repercussions on the reproductive function of the male gamete. To better characterize the GZ status of patients, we conducted an initial analysis that included a morphological and ultrastructural investigation by TEM to assess the proportion of round-headed spermatozoa. This is done to distinguish between the partial and complete forms of globozoospermia and therefore identify the best method of treatment. While our TEM results confirmed the diagnosis of round-headed spermatozoa and absent acrosome, we needed to investigate whether it was possible to treat these GZ men by confirming the integrity of the sperm genome as well as other sperm components such as the centrosome. The centrosome is needed to form the sperm aster, which is responsible for male and female centripetal pronuclear migration supporting the first mitotic spindle that can properly distribute chromosomes into the first two daughter cells [38, 39].

During spermatogenesis, an intricate process of sperm chromatin condensation is achieved by the replacement of histones with protamines, promoting the increased generation of disulfide bonds [40–43]. A disturbance in this mechanism leads to the production of spermatozoa with maturity defects and chromatin compaction abnormalities, resulting in a more fragile chromatin that is prone to damage from ROS and reduced decondensation ability during the pre-fertilization steps [44]. In our study, the aniline blue assessment of the residual histones revealed a higher percentage of spermatozoa with protamine deficiency in all GZ men, with a higher representation in the complete form as confirmed by other studies [45, 46]. This observation, however, did not reach statistical significance. An additional case study that used our chromatin compaction assay reported a histone content of 53.6% [47], very similar to our proportion in CG men.

It would be intuitive to assume that the incomplete compaction of the chromatin would lead to a higher frequency of SCF. Interestingly, however, the percentage of spermatozoa with chromatin fragmentation in our patients showed only a mild increase above threshold. Although one case study showed an SCF within the normal range in GZ patients [48], the large majority are in agreement with our findings, demonstrating an increased level of DNA fragmentation in a large cohort of 30 CG men [45, 47, 49–52].

Another aspect of the integrity of GZ spermatozoa revolved around the incidence of sperm aneuploidy that was higher in CG forms, as shown in another study [43], confirming the compromised meiotic spermatogenesis of these men in addition to the spermiogenic error [50, 53]. The inconsistent aneuploidy findings among GZ men may simply be related to the severity of globozoospermia or the screening tests used, such as FISH [54]. In this study, we assessed aneuploidy by 9-chromosome FISH as in prior reports [32], and then performed whole molecular karyotyping to validate the meiotic error and confirm an increased aneuploidy, particularly disomies, of chromosomes mostly involved in pregnancy loss.

We then focused on the presence of OASCF due to the absence of acrosome and perinuclear theca. These round-headed spermatozoa may be able to somewhat penetrate, if even, the outer layer of the *cumulous oophorous*, but even if they would be able to reach the surface of a denuded oocyte they would not penetrate the zona pellucida and are not able to fuse with the oolemma nor trigger oocyte activation. Insemination by ICSI enables the spermatozoa to bypass the barrier represented by the above-mentioned structures but cannot guarantee successful fertilization. To determine the best treatment method, we assessed OASCF by PLCζ content. When the presence of PLCζ was below 30%, the couple was treated by ICSI with AGT in their subsequent cycles [20]. Couples with a normal presence of OASCF were treated by conventional ICSI.

The PLCζ assay confirmed the presence of OASCF in the PG cohort, who were therefore treated in subsequent ICSI cycles that yielded a higher, albeit nonsignificant, clinical pregnancy and delivery rate. Inconsistent clinical outcomes reported in previous cases with this type of globozoospermia may be attributed to the fact that, during the ICSI procedure, it is possible to identify individual spermatozoa that have a somewhat physiological shape with residual acrosome and higher chromatin compaction (Supplementary Tables 1 & 2).

A more severe lack of PLCζ expression was observed in the CG group. This finding has also been reported previously [5] and more recently by our group [20]. These couples’ initial ICSI cycles resulted in a very slim fertilization rate that yielded no implantable embryos. Once treated by ICSI with AGT in subsequent cycles, however, they achieved a 37.5% fertilization rate that yielded a higher number of embryos available for transfer, resulting in a clinical pregnancy rate of 50% (Table 2).

Our genetic analysis of 3 CG men confirmed a deletion of 3 genes—DPY19L2, SPATA16, and PICK1—which are often associated with the globozoospermia phenotype and contribute to the most relevant feature of this condition, the absence of acrosome [55]. The encoded protein from DPY19L2 is situated in the nuclear membrane juxtaposed to the acrosomal vesicle and is responsible for securing the acrosome to the sperm nucleus [56]. An experiment on DPY19L2 knockout mice demonstrated arrested acrosome development and failed sperm head elongation, supporting these findings [57]. The deletion of this gene in humans is often associated with adequate sperm production, indicating a specific spermiogenic defect while having no effect on the proliferation of germ cells [58]. The deletion of SPATA16 is associated with acrosome biogenesis, occurring during the course of vesicle transport between the Golgi apparatus and pre-acrosomal granules, and has been confirmed in a homozygous point mutation (c.848G->A) [59] or a more extended deletion of 22.6 kb, which comprises the first coding exon [15]. Similarly, we found a deletion in the PICK1 gene, which also plays an important role in acrosome biogenesis [60].

In addition, we performed a genetic assessment according to the patients’ clinical outcomes. In the patient unable to generate an implantable embryo, a mutation on NLRP5, a gene responsible for the first zygotic division, was observed. A deletion of BSX, which is involved in neonatal development, was identified in the patient who had a pregnancy loss. For the patient who successfully achieved pregnancy, a single-nucleotide insertion in PIWIL1, responsible for frameshift mutation, was found to be limited exclusively to the characteristic sperm phenotype. PLCZ1, however, was mutated in all 3 CG patients, indicating that while the malfunction of this gene is related to the GZ phenotype, it did not necessarily affect the couples’ reproductive outcomes.

**Fig. 2:**
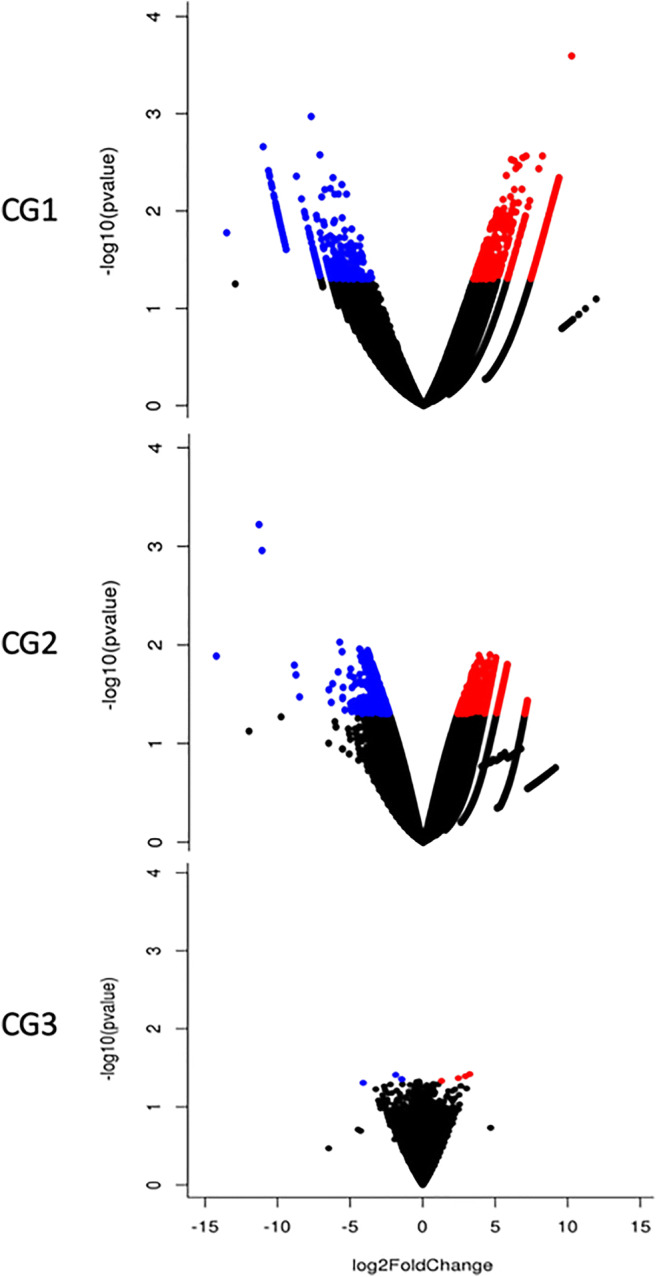
Global transcriptional change across the groups compared. All the genes are plotted, and each data point represents a gene. The log2 fold change of each gene is represented on the x-axis, and the log10 of its *P*-value is on the y-axis. Genes with a *P-value* < 0.1 and a log2 fold change greater than 1 are indicated by red dots, representing upregulated genes. Genes with a *P-value* < 0.1 and a log2 fold change less than 0 are indicated by blue dots, representing downregulated genes

We then further extended the transcriptome analysis and identified several imbalanced genes involved in reproductive function, solely in men unable to support a term pregnancy (Fig. 2). For instance, CG1, who failed to achieve implantation, had the highest number of imbalanced genes mainly involved in reproductive processes such as spermatogenesis and spermiogenesis. In particular, *STC2*, a gene responsible for embryo implantation, and *NEK2*, which is essential for centrosome separation, were highly downregulated (Supplementary Table 2). In CG2, whose female partner suffered a miscarriage at 8 weeks, RNA analysis of spermatozoa identified 3 imbalanced genes involved in important DNA-binding transcription factor activity (*RMI1*) and embryogenesis (*CBX2* and *TGFB3*) (Supplementary Table 3). The impaired expression of these genes may have therefore compromised the ability of spermatozoa from these men to support a term pregnancy [61]. Lastly, negligible gene imbalances were identified in CG3, whose partner reported a successful term pregnancy. Overall, while these results add to previous findings that identified significantly lower PLCζ expression in infertile globozoospermic patients [12], they need to be confirmed in a larger sample size to identify robust relationships between transcriptomic profiles and reproductive outcome.

The mutations and expression profiles of RNA and proteins corresponded well, as was the case for HIST1H1B, DPY19L2, BAG5, and SPACA4. Other proteins, however, exhibited a paradoxical profiling. In particular, RCC1 and H1FNT both displayed underexpressed RNA and protein profiles, despite a mutation of single-nucleotide insertion on the corresponding genes. It is therefore possible that these mutations cause a premature stop codon, which has been shown to result in production of a truncated protein due to nonsense mediated decay [25].

While these findings demonstrate that gene expression can provide essential information about the reproductive capacity of spermatozoa, this study is not without limitations. There was a small number of patients enrolled, which can be due to the rare incidence of globozoospermia within the infertile population. However, unlike most of the current literature on globozoospermia that involve case or multi-centered studies, ours is the largest study performed by a single ART center [22]. Nevertheless, these results should be confirmed in a larger study population.

## Conclusions

Our study aimed to investigate the male gamete of GZ men through multifaceted assessments to acquire specific knowledge about their infertility, particularly their PLCζ level, to guide treatment decisions for enhanced reproductive outcomes. We found that the use of AGT can overcome a low fertilization rate or complete fertilization failure in the absence of OASCF. However, the condition of globozoospermia is not limited to missed acrosomal migration; it can also affect chromatin compaction, which may interfere with sperm chromatin stability and integrity, and eventually the chromosomal constitution of the male genome resulting from meiotic abnormalities. Therefore, elucidating the genetic and epigenetic content of GZ men can shed light on the spermatogenic process and the gamete’s capacity to support embryonic development and implantation.

## Supplementary information

ESM 1 (PNG 158 kb)

ESM 2 (PNG 145 kb)

ESM 3 (PNG 165 kb)

ESM 4 (DOCX 25 kb)
