# Supplementary figures and images for: Single-center thorough evaluation and targeted treatment of globozoospermic men
Source: J Assist Reprod Genet. 2021 Apr 20;38(8):2073–86. doi: 10.1007/s10815-021-02191-4 (PMC8417186; doi:10.1007/s10815-021-02191-4)

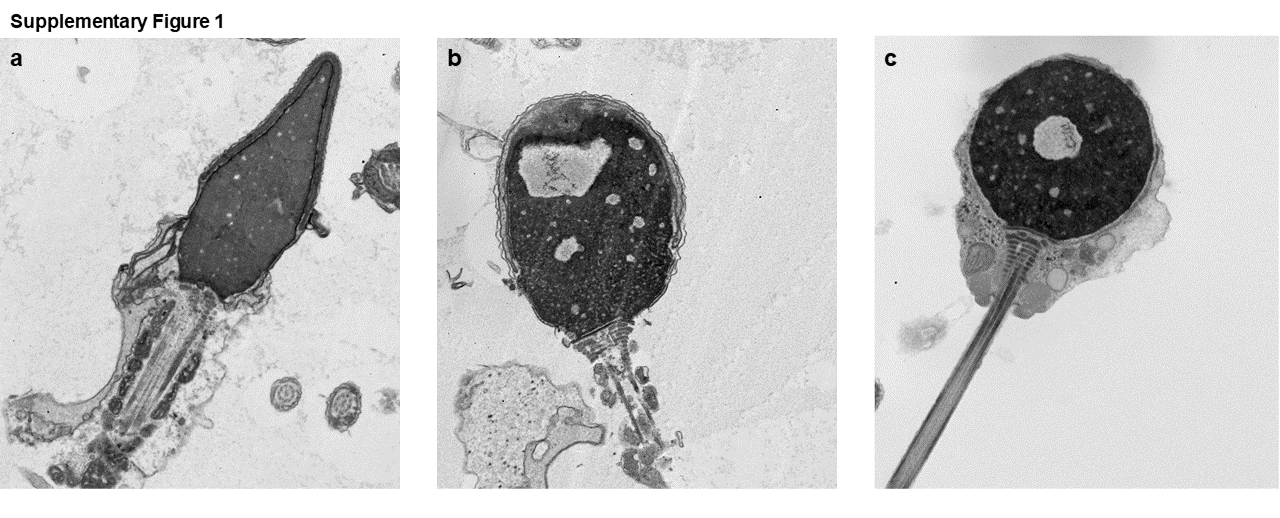

Supplement: Supplementary file 1 — (PNG 158 kb) [file 10815_2021_2191_MOESM1_ESM.png]

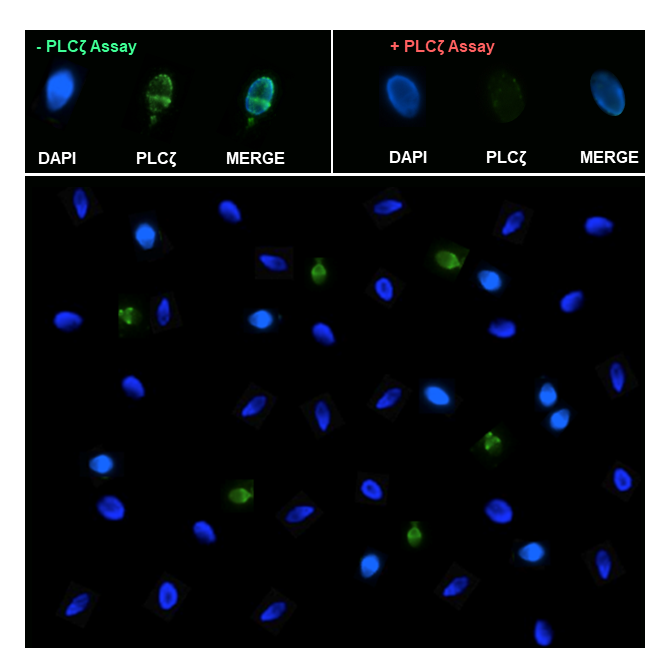

Supplement: Supplementary file 2 — (PNG 145 kb) [file 10815_2021_2191_MOESM2_ESM.png]

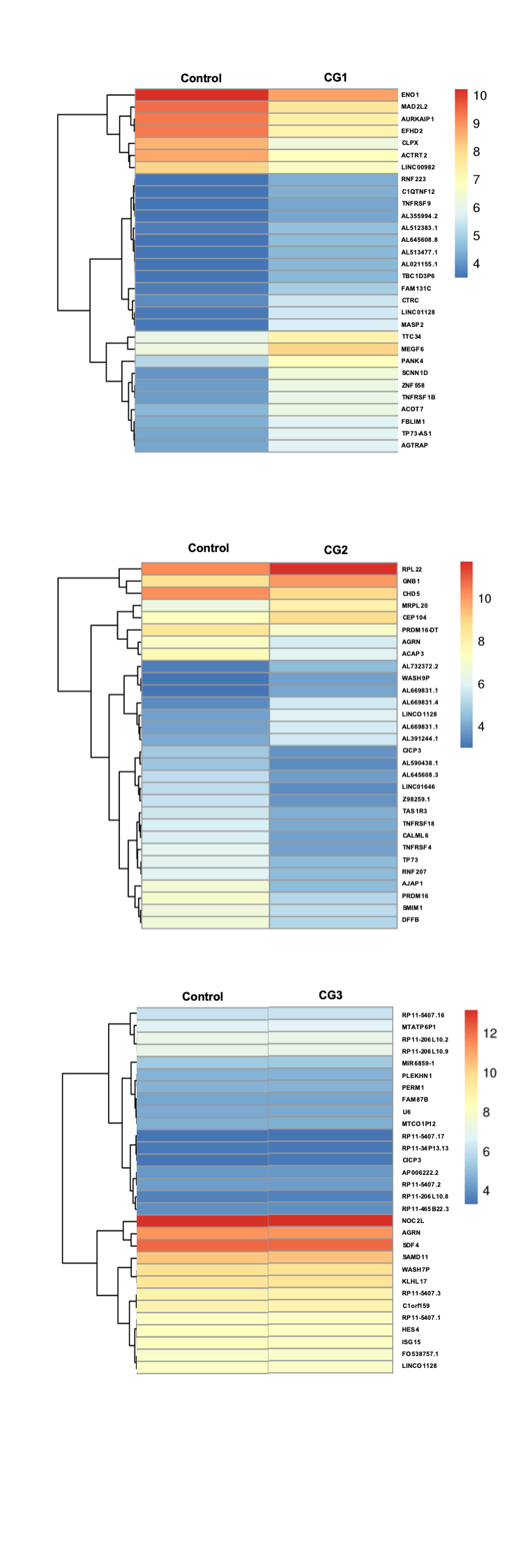

Supplement: Supplementary file 3 — (PNG 165 kb) [file 10815_2021_2191_MOESM3_ESM.png]
