# Supplementary material for: Single-center thorough evaluation and targeted treatment of globozoospermic men
Source: J Assist Reprod Genet. 2021 Apr 20;38(8):2073–86. doi: 10.1007/s10815-021-02191-4 (PMC8417186; doi:10.1007/s10815-021-02191-4)
Supplement: Supplementary file 4 — (DOCX 25 kb) [file 10815_2021_2191_MOESM4_ESM.docx]

**Supplementary Figure Legends:**

**Supplementary Figure 1.** Ultrastructural details by TEM: a) a spermatozoon from fertile donor with normal acrosome and compact nucleus (20,000x magnification); b) a spermatozoon from a patient with partial globozoospermia (20,000x magnification); c) a round-headed spermatozoon from CG1 with abnormal nuclear condensation, vacuolization, cytosolic residual, and absence of acrosome (20,000x magnification)

**Supplementary Figure 2.** Immunofluorescence staining for PLCζ: Spermatozoa were visualized under a fluorescent microscope to assess the percentage of spermatozoa exhibiting PLCζ fluorescence in the acrosomal, equatorial, and post-acrosomal regions of the sperm head. By assessing a large number of patients for PLCζ, we found that those with less than 30% PLCζ yielded consistently low or unobtainable fertilization. Therefore, a normal threshold of >30% was used.

**Supplementary Figure 3.** Differentially expressed genes bi-clustering heat map: This analysis is performed to visualize the expression profile of the 30 most imbalanced genes sorted by their adjusted *P* value. This analysis is useful to identify co-regulated genes across the treatment conditions.

**Supplementary Tables:**

**Supplementary Table 1.** Semen Parameters and Specific Abnormal Morphology of all Patients and According to Globozoospermia Type

| **Parameters (mean ± SD)** | **All** | **Partial** | **Complete** | ***P* Value** |
| --- | --- | --- | --- | --- |
| **Patients** | 14 | 4 | 10 |  |
| **Volume (mL)** | 2.4 ± 0.7 | 2.6 ± 1 | 2.5 ± 1 | NS |
| **Concentration (x10^6^ /mL)** | 36.0 ± 44 | 31 ± 42 | 36.5 ± 39 | NS |
| **Total Motility (%)** | 20.3 ± 20 | 15 ± 12 | 24.4 ± 21 | NS |
| **Normal Morphology (%)** | 0.2 ± 0.4 | 0.7 ± 0.5 | 0 ± 0 | 0.003 |
| **Abnormal Morphology Distribution** | | | | |
| **Heads (%)** | 87.5 ± 31 | 73.5 ± 49 | 100 ± 0 | NS |
| **Neck (%)** | 1.1 ± 2 | 1 ± 2 | 0 | NS |
| **Tail (%)** | 1.3 ± 4 | 0 ± 0 | 0 | NS |
| *P*<0.05 was considered to be statistically significant | | | | |

**Supplementary Table 2.** Significantly Underexpressed Genes Involved in Reproductive Processes Found in Patient CG1 as Compared to a Known Fertile Control

|  |  | **Log2 Fold Change** | |  |
| --- | --- | --- | --- | --- |
| **Gene** | **Chr** | **Transcriptomics** | **Proteomics** | **Description** |
| *DPY19L2* | 12 | -6.53 | -6.13 | Spermiogenesis, head elongation, acrosome development |
| *UBR2* | 6 | -4.81 | 2.5 | Spermatogenesis |
| *TXNRD3* | 3 | -4.50 | -2.3 | Sperm maturation |
| *NEK2* | 1 | -3.79 | 3.5 | Centrosome separation, polar spindle formation in mitotic cells, chromatin condensation in meiotic cells |
| *KLHDC3* | 6 | -3.53 | -3.5 | Meiotic recombination |
| *H1FNT* | 12 | -3.30 | -8.4 | DNA condensation during spermiogenesis. Histone-protamine transition |
| *MFSD14A* | 1 | -3.22 | -3.2 | Globozoospermia, Spermatogenic failure |
| *ADAM21* | 14 | -3.16 | -3.2 | Sperm maturation and/or fertilization |
| *KLHL10* | 17 | -3.01 | -1.4 | Associated with oligozoospermia, male infertility |
| *STC2* | 5 | -3.00 | 5.8 | Embryo implantation |
| *HSPA1L* | 6 | -2.97 | -1.0 | Heat shock protein, fertilization, embryogenesis |
| *RNF8* | 6 | -2.90 | -2.3 | Replacement of histones during spermatogenesis |
| *PAPPA* | 9 | -2.87 | 2.4 | Encodes a secreted metalloproteinase which cleaves insulin-like growth factor binding proteins (IGFBPs); female infertility |
| *CNBD2* | 20 | -2.79 | -2.8 | Spermatogenesis, development of the flagellar |
| *MORN2* | 2 | -2.74 | -2.8 | Spermatogenesis |
| *IQCF1* | 3 | -2.73 | -2.7 | Sperm capacitation and acrosome reaction |
| *PICK1* | 22 | -2.5 | -3.4 | Sperm acrosome formation |
| *OVGP1* | 1 | -2.49 | 5.0 | ZP binding, fertilization and/or early embryonic development. |
| *CALR3* | 19 | -2.48 | -2.5 | Calcium ion binding, required for fertilization |
| *WNT9B* | 17 | -2.48 | 6.9 | Embryo development |
| *SPEM1* | 17 | -2.32 | -2.5 | Required for proper cytoplasm removal during spermatogenesis; Spermatogenic failure |
| *SPATA16* | 3 | -1.4 | -2.2 | Acrosome formation, spermatogenesis, and sperm-egg fusion |

**Supplementary Table 3.** Significantly Underexpressed Genes Involved in Reproductive Processes Found in Patient CG2 as Compared to a Known Fertile Control

| **Gene** | **Chr** | **Log2 Fold Change** | **Description** |
| --- | --- | --- | --- |
| *SMCP* | 1 | -10.6 | Organization of sperm mitochondria, associated with sperm motility and zona pellucida penetration |
| *NAMPT* | 7 | -9.73 | Spermatogenesis |
| *SEMG2* | 20 | -7.76 | Male infertility |
| *GATA1* | X | -6.68 | DNA-binding transcription factor activity and chromatin binding |
| *CRHR1* | 17 | -6.35 | Promotes the activation of adenylate cyclase, embryonic development of the adrenal gland |
| *RMI1* | 9 | -5.8 | DNA double-strand break repair |
| *FOXL2* | 3 | -5.69 | DNA-binding transcription factor activity, transcriptional repression of the Sertoli cell-promoting gene SOX9 |
| *ACVR1C* | 2 | -5.55 | Cell differentiation, growth arrest, apoptosis, embryogenesis |
| *SEMG1* | 20 | -4.88 | Male infertility, human semen coagulum, regulates sperm progressive motility |
| *LHX9* | 1 | -4.83 | Sequence-specific DNA binding and transcription corepressor activity, gonal development |
| *CSF1* | 1 | -4.39 | Required for normal male and female fertility, reorganization of the actin cytoskeleton, formation of membrane ruffles, cell adhesion and cell migration |
| *TGFB3* | 14 | -4.09 | Embryogenesis and cell differentiation |
| *CBX2* | 17 | -3.91 | Chromatin binding and methylated histone binding; embryo development. |

**Supplementary Table 4.** Common Gene Imbalances Between Patients Unable to Sustain a Term Pregnancy (CG1 and CG2) as Compared to a Known Fertile Control

| **Gene** | **Chr** | **Description** |
| --- | --- | --- |
| *HBB* | 11 | Involved in oxygen transport |
| *HBA1* | 16 |  |
| *HBA2* | 16 |  |
| *NT5DC1* | 6 | Hydrolase and 5’-nucelotidase activity, associated with Metaphyseal Chondrodysplasia |
| *WBP2* | 17 | Transcriptional coactivator of estrogen and progesterone receptors |
| *NME4* | 16 | Synthesis of nucleoside triphosphates |
| *PHACTR4* | 1 | Regulator of protein phosphatase 1 |
| *LYL1* | 19 | Basic helix-loop-helix transcription factor, blood vessel maturation and hematopoiesis |
| *DMRTA1* | 9 | Oxygen transport DNA-binding transcription factor activity and sequence-specific DNA binding |
| *FAM50A* | X | DNA-binding protein, transcriptional factor |
| *GRHL2* | 8 | DNA-binding transcription factor activity and chromatin DNA binding |
| *MED1* | 17 | Chromatin binding |
| *NEK2* | 1 | Chromatin condensation in meiotic cells, centrosome separation and bipolar spindle formation in mitotic cells |
| *UBR2* | 6 | Spermatogenesis, chromosome-wide transcriptional silencing during meiosis via ubiquitination of histone H2A |

**Supplementary Table 5.** Genomic and Proteomic Analyses for Complete Globozoospermic Men as Compared to a Known Fertile Control

|  |  |  | **Log2 Fold Change** | |  |
| --- | --- | --- | --- | --- | --- |
| **Gene** | **Chr** | **Mutation** | **Transcriptomics** | **Proteomics** | **Description** |
| *RCC1* | 1 | Duplication | -2.30 | -6.62 | Regulator of chromosome condensation, chromatin and nucleosomal DNA binding |
| *HIST1H1B* | 6 | Deletion | -3.12 | -3.98 | Condensation of nucleosome chains into higher-order structured fibers |
| *DPY19L2* | 12 | Deletion | -6.53 | -6.13 | Spermiogenesis, head elongation, acrosome development |
| *H1FNT* | 12 | Duplication | -3.30 | -8.35 | Essential for spermatogenesis and male fertility; required for proper cell restructuring and DNA condensation during elongation phase of spermiogenesis; involved in histone-protamine transition of sperm chromatin and subsequent production of functional spermatozoa; binds double- and single-stranded DNA, ATP and protamine-1 |
| *BAG5* | 14 | Deletion | -2.41 | -4.24 | Cell apoptosis |
| *SPACA4* | 19 | Deletion | -2.05 | -9.07 | Sperm-egg membrane adhesion and fusion during fertilization |

**Supplementary Table 6.** Localization, Type, and Minor Frequency Allele (MAF) of Main Mutations Identified in Complete Globozoospermic Men as Compared to a Known Fertile Control

| **Gene** | **Type** | **Previously described** | **Location** | **MAF** |
| --- | --- | --- | --- | --- |
| DPY19L2 | Deletion | No | c.211_*29del | n/a |
| SPATA16 | Deletion | No | c.*620del | 0.0517 |
| PICK1 | Deletion | No | n.38478847_38369512del | n/a |
| PIWIL1 | Insertion | No | n.-11993_*42972dup | n/a |
| NLRP5 | Insertion | No | c.2630_3128+149dup | n/a |
| BSX | Deletion | No | n.122968770_122852254del | n/a |
| PLCZ1 | Insertion | No | n.18719878_19353403dup  n.-462457_*168820dup  n.-485722_*129206dup | n/a |
